# Pathological Features and Genetic Polymorphism Analysis of Tomato Spotted Wilt Virus in Infected Tomato Fruit

**DOI:** 10.3390/genes14091788

**Published:** 2023-09-12

**Authors:** Junheng Lv, Yunrong Mo, Minghua Deng, Junqiang Xu, Bin Xu, Xinyun Li, Jing Li, Caiqian Jiang, Ying Zhou, Ziran Wang, Zhengan Yang, Kai Zhao

**Affiliations:** 1 College of Landscape and Horticulture, Yunnan Agricultural University, Kunming 650201, China; 2 College of Food Science and Technology, Yunnan Agricultural University, Kunming 650201, China

**Keywords:** tomato, tomato spotted wilt virus, pathological features, polymorphism analysis, resistance breaking

## Abstract

An in-house tomato inbred line, YNAU335, was planted in a greenhouse in spring from 2014 to 2017, and showed immunity to tomato spotted wilt virus (TSWV). YNAU335 was infected with TSWV in the spring from 2018 to 2020, and disease was observed on the leaves, sepals, and fruits. In 2021 and 2022, YNAU335 was planted in spring in the same greenhouse, which was suspected of being infected with TSWV, and visible disease symptoms were observed on the fruits. Transmission electron microscopy, deep sequencing of small RNAs, and molecular mutation diagnosis were used to analyze the pathological features and genetic polymorphism of TSWV infecting tomato fruit. Typical TSWV virions were observed in the infected fruits, but not leaves from YNAU335 grown between 2021 and 2022, and cross-infection was very rarely observed. The number of mitochondria and chloroplasts increased, but the damage to the mitochondria was greater than that seen in the chloroplasts. Small RNA deep sequencing revealed the presence of multiple viral species in TSWV-infected and non-infected tomato samples grown between 2014–2022. Many virus species, including TSWV, which accounted for the largest proportion, were detected in the TSWV-infected tomato leaves and fruit. However, a variety of viruses other than TSWV were also detected in the non-infected tissues. The amino acids of TSWV nucleocapsid proteins (NPs) and movement proteins (MPs) from diseased fruits of YNAU335 picked in 2021–2022 were found to be very diverse. Compared with previously identified NPs and MPs from TSWV isolates, those found in this study could be divided into three types: non-resistance-breaking, resistance-breaking, and other isolates. The number of positive clones and a comparison with previously identified amino acid mutations suggested that mutation F at AA118 of the MP (GenBank OL310707) is likely the key to breaking the resistance to TSWV, and this mutation developed only in the infected fruit of YNAU335 grown in 2021 and 2022.

## 1. Introduction

Tomato spotted wilt virus (TSWV) is an important virus in the genus *Orthotospovirus*, family *Tospoviridae*, and order *Bunyavirales*. Listed as one of the ten most harmful plant viruses in the world, it is the only genus with plant-infecting members within the family *Bunyaviridae* [1,2]. TSWV was first reported in Australia and quickly spread throughout the world. The host range of TSWV exceeded 1000 plants, including many horticultural crops of *Solanaceae* [3,4]. China is one of the most important tomato production areas in the world, and TSWV infections have occurred frequently and have caused significant losses in recent years. Necrosis, verticillium wilt, and spots on the leaves, stems, and fruits were often observed in the infected plants. The diseased spots were serious when the fruit was fully mature and had a strong impact on the tomato yield and fruit quality [5].

TSWV disease-resistant tomato resources were distributed mainly in wild tomatoes. In particular, Peruvian and Chilean tomatoes represented the most comprehensively resistant resources in which a number of excellent disease-resistant tomato materials and genes were identified [6,7]. Multiple TSWV-resistant genes, namely *Sw-1a*, *Sw-1b*, *Sw-2*, *Sw-3*, *Sw-4*, *Sw-5a*, *Sw-5b*, *Sw-6*, and *Sw-7*, were derived from common tomato (*Solanum lycopersicum* L.) or wild tomato plants [8,9,10,11]. The resistance to TSWV conformed to a single-gene dominant inheritance model and was controlled by a dominant quality gene, *Sw-5* [11,12]. Multiple molecular markers, such as RAPDs, RFLPs, and SCARs, were developed for *Sw-5* [13,14,15]. Two CC-(NB-ARC)-LRR genes, *Sw5-a* and *Sw5-b*, which were located in the *Sw-5* locus, were cloned, but only *Sw5-b* increased resistance to TSWV [16].

Under an electron microscope, typical quasi-spherical and dumbbell-shaped particles of 80–100 nm in diameter were found in cells infected with TSWV [17,18]. The accumulation of *Tospovirus* in the endoplasmic reticulum pool in the host cytoplasm was the most important cytopathological feature of this virus genus [19]. TSWV, a representative of the genus *Tospovirus*, was observed to have this feature in infected tomato fruits [18]. Phytopathological changes in the host cells infected by TSWV included vacuoles in chloroplasts and mitochondria, as well as the destroyed lamella structure of chloroplasts.

Replicating viral RNA genomes into small interfering RNAs (siRNAs) of discrete sizes were processed to guide virus clearance by RNA interference [19]. It was hypothesized that it would be possible to identify viral species infecting different crops using deep sequencing of siRNAs [20]. Deep sequencing technologies facilitated the identification of numerous viruses and novel viruses, such as the Grapevine Pinot gris virus and rose leaf rosette-associated virus [21,22,23].

The genome of TSWV comprised a negative single-stranded RNA molecule consisting of three parts: L RNA, M RNA, and S RNA, and containing five open-reading frame coding for four structural and two non-structural proteins [24]. Latham and Jones [25] selected TSWV isolates and inoculated tomatoes containing the *Sw-5* gene, with the results indicating that a small number of tomatoes not only had local allergic reactions but also showed susceptibility in the whole plant, suggesting that some TSWV isolates had overcome the *Sw-5* gene-based resistance. Subsequently, other breaks in *Sw-5* resistance were observed [26,27,28,29]. The primary determinant in overcoming resistance to these TSWV isolates was not the nucleocapsid protein (NP) gene but rather the movement protein (MP) gene [26]. It was found that C118 to Y118 and T120 to N120 together resulted in overcoming *Sw-5* resistance [29].

In this study, the tomato inbred line YNAU335, which previously showed immunity to TSWV, was investigated and demonstrated not to have the *Sw-5* gene [30]. YNAU335 was planted for three years from 2018 to 2020, during which time its resistance to TSWV was overcome. Disease occurred mainly in the leaves, sepals, and fruits. Gene polymorphism analysis of the MP and NP of TSWV showed that four specific mutations may be related to the break in YNAU335 resistance [31]. In 2021 and 2022, the inbred line, YNAU335, was suspected of being infected with TSWV, with the disease only being observed in the fruit. Through electron microscopy, virus-derived, small interfering RNA sequencing and molecular mutation diagnosis were used to systematically analyze the characteristics of TSWV. These results could provide a foundation for the analysis of the mechanisms of tomato TSWV resistance in the future.

## 2. Materials and Methods

### 2.1. Tomato Material

The inbred line, YNAU335, is the cultivated species (*Solanum lycopersicum* L.) that shows the characteristics of typical cultivated species, collected from Yuanmou County, Yunnan Province, China, and was used in this study. Leaves and fruits of YNAU335 were sampled annually from 2014 to 2022.

### 2.2. Virus Morphology Diagnosed

Infected tomato fruits of YNAU335 from 2021 to 2022 were chosen for negative staining. The parts ~0.2 cm below the tomato fruit surface were chopped into smaller pieces before being fixed with 2.5% glutaraldehyde. Transmission film was placed on the samples to absorb for 3–5 min, dried with filter paper, and placed on a surface with the virus facing upwards and air dried for 1 min. Two percent of ammonium molybdate (pH 5.5) was added to the dried films for 3 min and was dried to dye the virus material, which was then observed under a JEM100CX-II transmission electron microscope (JEOL Ltd., Tokyo, Japan) [32].

### 2.3. Ultra-Thin Section Sample Preparation and Observation

The parts ~0.2 cm below the tomato fruit surface were cut into small pieces of 1 mm × 1 mm × 3 mm and fixed in 2.5% glutaraldehyde solution for 12 h at 4 °C. A phosphate-buffered solution (0.2 mol/L, pH 7.2) was used to rinse the sample, which was then fixed in 1% OsO_4_ solution at 20 °C for 2 h. After ethanol gradient dehydration, epoxy resin Ep812 embedding, AO ultrathin microtome sectioning, and staining with 5% lead citrate and 1% uranyl acetate, the samples were observed and photographed under a JEM100CX-II transmission electron microscope (JEOL Ltd., Tokyo, Japan) [33].

### 2.4. Reverse Transcription-Polymerase Chain Reaction (RT-PCR)

TSWV, tomato zonate spot virus (TZSV) and tomato brown rugose fruit virus (ToBRFV) display similar symptoms in tomato fruits, and the virions of TZSV and ToBRFV are similar in shape. RT-PCR was used to further verify the virus species. The total RNA of fruits and leaves of YNAU335 of 2021–2022 was extracted using the Quick RNA Isolation Kit (HuaYueYang Biotech Co., Ltd., Beijing, China), and then treated with RNase-free Dnase I (Takara Biotechnology Co., Ltd., Dalian, China) to remove the genomic DNA. Total RNA (2 μg) was reverse transcribed into first-strand cDNA using the M-MLV Reverse Transcriptase Kit (Takara Biotechnology Co., Ltd., Dalian, China), according to the manufacturer’s protocol, and oligo (dT) primer and random primers were used in the reverse transcription reactions. The cDNA samples were used as templates for RT-PCR. Primers pMP, pNP, pTZSV, and pToBRFV for the TSWV-MP, TSWV-NP, TZSV, and ToBRFV, respectively, are shown in Table S1.

PCR reaction system: 25 μL of PCR high-fidelity enzyme (Takara Biotechnology Co., Ltd., Dalian, China), 1 μL each of the upstream and downstream primers, 1 μL of cDNA template, and sterile ddH_2_O were combined to yield a final volume of 50 μL. PCR conditions: pre-denaturation at 98 °C for 2 min, followed by denaturation at 98 °C for 10 s and annealing for 5 s, with the annealing temperature set according to the base composition of each primer. Extension was performed at 72 °C for 1 min for 40 cycles. The final extension lasted 5 min.

### 2.5. RNA Isolation, Library Preparation, and Sequencing

Three grams of fresh material was sent to Shanghai Majorbio Bio-pharm Technology Co., Ltd. (Shanghai, China) for deep sequencing. Total RNA was spliced at the 5′ and 3′ ends using a Truseq^TM^ Small RNA Sample Prep Kit. First-strand cDNA was obtained via reverse transcription with random primers using the Truseq^TM^ Small RNA sample prep Kit. The library was enriched using PCR for 11 to 12 cycles. The product was recovered using PAGE electrophoresis and quantitated on a TBS380 (Invitrogen, Carlsbad, CA, USA). The clusters were generated using the cBot system and sequenced on an Illumina HiSeq 2000 platform [34]. The raw sequencing data were deposited in the NCBI Short Read Archive (SRA, BioProject ID: PRJNA673290).

### 2.6. Read Assembly and Library Bioinformatics Analysis

The raw sequencing data of the fruits and leaves of YNAU335 planted in 2014–2017, 2018–2020 and 2021–2022 were assessed for quality and subjected to data filtering using the Fastx-Toolkit program (http://hannonlab.cshl.edu/fastx_toolkit/, (accessed on 11 November 2022)). After quality control, the high-quality sequencing reads were assembled de novo to generate contigs and singletons, and the small RNAs that ranged from 18 to 40 nt were also counted. The assembled data were compared with the virus database (http://www.dpvweb.net/, (accessed on 22 November 2022)) and annotated. According to the results of the assembly and comparison with the plant virus database, the virus with the highest proportion was selected, and the corresponding viral genome sequence was downloaded from NCBI to predict the virus-induced siRNA.

### 2.7. Polymorphism Analysis of the NP and MP Genes of TSWV

The fruits of infected tomatoes in 2021 and 2022 were used as experimental materials. Sample RNA extraction refers to the above-mentioned method. Forty positive colonies were randomly selected to identify the NP and MP genes of TSWV. The colonies were cultured for development and identification, followed by sequencing at Beijing Tsingke Biotech Co., Ltd. (Beijing, China). The gene accession number of the sequenced and spliced coding sequence was obtained through the BankIt submission system at https://www.ncbi.nlm.nih.gov/, (accessed on 25 November 2022). MEGA5.0 was used to analyze the amino acid mutation sites on the NP and MP.

## 3. Results

### 3.1. Symptoms of Infected Tomato Fruit

**Figure 1:**
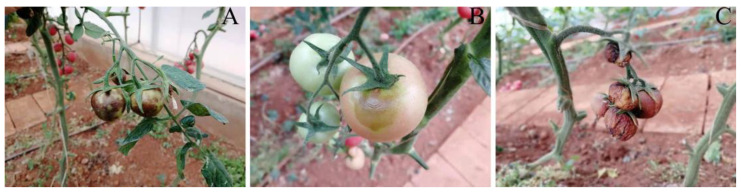
Symptoms of diseased fruit of YNAU335 planted in 2021 and 2022. (**A**) green fruit; (**B**) fruit during the color-changing period; (**C**) infected fruit.

Throughout the growth period from 2021 to 2022, the tomato leaves of YNAU335 were normal in appearance and showed no symptoms of TSWV. The green fruit showed chlorotic ring spots, tumor-like protrusions, and blurred ring patterns (Figure 1A). During the ripening period, the fruit showed obvious diseased ring patterns (Figure 1B). Diseased fruits did not expand normally, with the whole fruit exhibiting a red color and collapsed shape (Figure 1C).

### 3.2. Virus Morphology Diagnosis by Negative Staining

**Figure 2:**
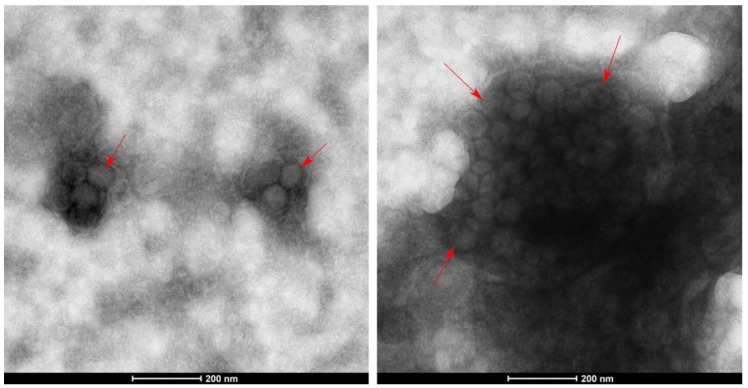
Morphological characteristics of tomato spotted wilt virus in infected fruits of YNAU335 planted in 2021 and 2022 using negative staining technique. The red arrows indicate the typical TSWV virions.

In order to preliminarily identify the species of virus infecting tomato fruit of YNAU335 planted in 2021 and 2022, a negative staining technique was used to analyze the virus. Typical TSWV virions, with a diameter of ~80 nm and aggregates with a nearly spherical shape, were observed (Figure 2).

### 3.3. Pathological Features of Tomato Fruit Cells Based on Ultramicrotomy

**Figure 3:**
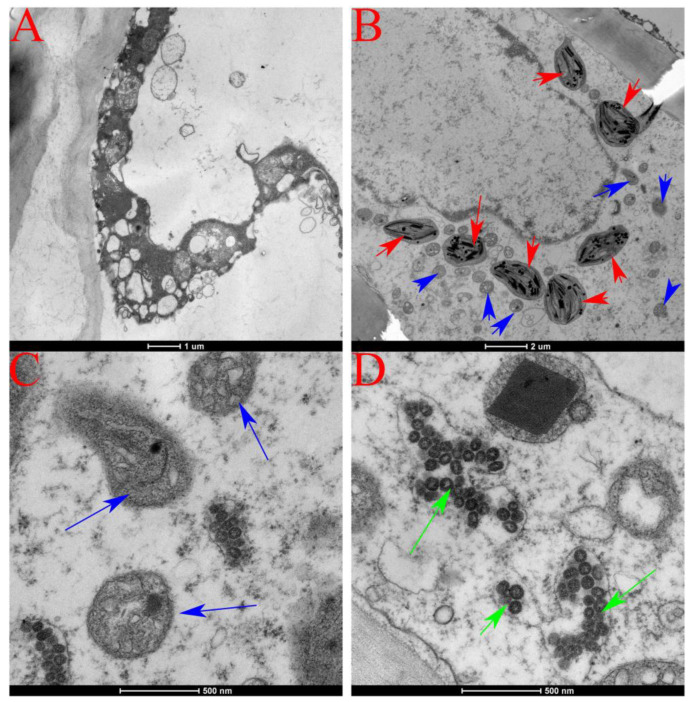
Ultrastructural changes of infected tomato fruit cells of YNAU335 planted in 2021 and 2022 using ultramicrotomy technique. (**A**) pathological characteristics of diseased cells; (**B**) increase in the number of mitochondria (blue arrows) and chlorophyll (red arrows); (**C**) mitochondrial structural changes; (**D**) tomato spotted wilt virus particles (green arrows).

The infected tomato fruits showed severe subcellular pathological changes with plasmolysis observed. The nucleus, cell membrane, endoplasmic reticulum, Golgi apparatus, and other substructures disappeared, with only the cell wall, mitochondria, chloroplasts, vacuoles, and peroxisomes observed (Figure 3A). The number of vesicles, mitochondria and chloroplasts in the cells increased, and the damage to the mitochondria was greater than that seen in the chloroplasts. The matrix lamella structure was relatively intact, and only a few lamellar structures had disintegrated (Figure 3B). Vesicle formation occurred inside the mitochondria, and parts of the mitochondrial structures collapsed (Figure 3C). TSWV viral particles were observed to be distributed in the diseased cells (Figure 3D).

### 3.4. Identification of the Types of Viruses Using RT-PCR

**Figure 4:**
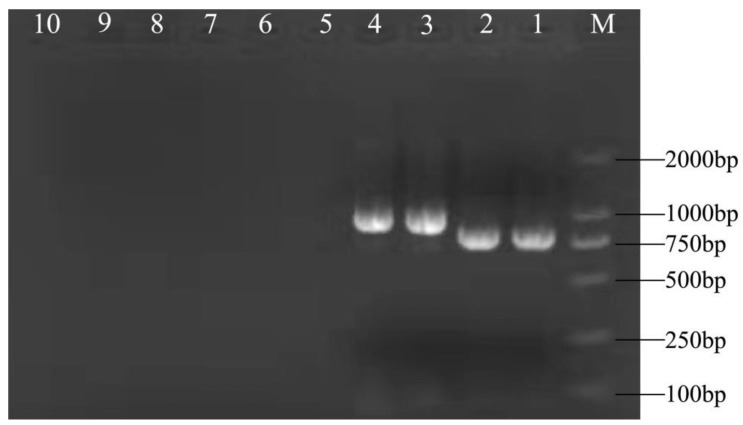
RT-PCR identification of the virus types infecting the fruit of YNAU335 planted in 2021 and 2022. M: DNA Marker 2000; Lanes 1 and 2: TSWV NP genes in fruit in 2021 and 2022; Lanes 3 and 4: TSWV MP genes in fruit in 2021 and 2022; Lanes 5 and 6: TSWV NP genes in leaves in 2021 and 2022; Lanes 7 and 8: TZSV genes in fruit in 2021 and 2022; Lanes 9 and 10: ToBRFV genes in fruit in 2021 and 2022.

TSWV, TZSV, and ToBRFV display similar symptoms in tomato fruits, and it is difficult to distinguish them based on symptoms alone. RT-PCR was used to determine which virus the tomato fruit was infected with. The results showed that the NP and MP genes of TSWV were cloned from the fruits of the infected tomatoes of 2021 and 2022 (Figure 4, Lane 1–4), but not the leaves (Figure 4, Lane 5–6). However, TZSV and ToBRFV gene fragments were not cloned from the tomato fruits in 2021 and 2022 (Figure 4, Lane 7–10).

### 3.5. Small RNA Sequencing and Virus Species Identification

From 2014 to 2017, the YNAU335 inbred line showed immunity to TSWV. High-throughput, small RNA sequencing showed that TSWV was not detected in fruit and leaves, and the species and amount of viruses in fruit and leaves were similar. Pepper chlorotic spot virus, tobacco vein clearing virus and southern tomato virus were the only plant viruses found. Most of the others were bacterial and animal viruses, with the largest number of bacterial viruses, which included seven in fruits and leaves (Tables S2 and S3). From 2018 to 2020, the resistance of YNAU335 was broken, and a large amount of TSWV was detected in infected fruits and leaves. TSWV in fruits and leaves accounted for 74.53% and 49.69%, respectively, and ranked first among all virus species. There were ten and nine kinds of plant viruses identified in fruits and leaves, accounting for 99.23% and 90.89%, respectively. The nine kinds of viruses in leaves were the same as those in fruits (Tables S4 and S5). From 2021 to 2022, 25 kinds of viruses were detected in YNAU335-infected fruits, among which 11 kinds of plant viruses, including TSWV, accounted for 68.44%, and the second plant virus Tomato chlorotic spot virus accounted for only 5.75% (Table S6). In total, sixteen kinds of viruses were detected in leaves, among which only three kinds of plant viruses were detected, which did not include TSWV (Table S7).

### 3.6. Polymorphism Analysis of TSWV Movement Proteins (MPs) and Nucleocapsid Proteins (NPs) in Diseased Tomato Fruits

Three previously submitted MPs (MK883723, MK883724, and MK887284) and five NPs (MK628735, MK628736, MK628737, MK628738, and MK628739) were selected as controls [31]. The sequence analysis from TSWV-infected fruit of YNAU335 in 2021–2022 showed that there were more positive clone variations in the NP (11) than in the MP (7); moreover, amino acid mutations in the NP (13) were more numerous than those in the MP (10) (Table 1 and Table 2). Among the MP mutations, two amino acid sites were found to have a higher mutation frequency, namely F118 and M201. Compared with the three previously submitted MP mutations, F118 represented a new amino acid mutation site (Table 1). The specific amino acid mutation sites of NP were the same as those for MK628735 and MK628736. Another 11 mutations were observed, but each of these mutated amino acids existed only in one positive clone (Table 2).

## 4. Discussion

In each spring from 2014 to 2022, three inbred tomato lines, No. 5, CLN2037E, and 96172I in a greenhouse planted with YNAU335, were infected with TSWV every year, or even failed to harvest. Therefore, YNAU335 was guaranteed to be in a TSWV-susceptible environment every year. After small RNA deep sequencing, abundant virus species were detected in infected and non-infected YNAU335 plants. TSWV was not detected in YNAU335 plants showing no symptoms of TSWV infection. Most of the detected viruses were bacteriophage viruses and a small amount were animal viruses. The number of TSWV sequences in TSWV-infected tomato tissues accounted for more than 50% of all virus sequences. However, other kinds of viruses were present in TSWV-infected tissues that were roughly the same as those in non-infected plants. The exclusion of these same viruses indicated that TSWV played a dominant role in YNAU335 infection.

The accumulation of *Tospovirus* in the endoplasmic reticulum pool in the host cytoplasm is the most important cytopathological feature of the genus virus [19]. TSWV, as a representative of *Tospovirus*, was also observed with this feature in the infected tomato fruits in this study. Under natural conditions, the mixed infection of two or more viruses on the same host plant is quite common. Mixed infection caused by different viruses were antagonism or synergism [35]. TSWV and the tomato chlorosis virus, and TSWV and the iris yellow spot virus, showed synergism in mixed infection of tomato and *Datura stramonium* [36,37]. There is no synergism observed in mixed infection of TSWV and Potato Virus Y in peanuts [38]. Zhang et al. (2004) found that there may be antagonism in mixed infection of TSWV and tobacco mosaic virus in *Cymbidium hybridum* [39]. This study showed that TSWV and tomato chlorotic spot virus proliferated in the cells of the host, with very little cross-infection, indicating that antagonism may exist when TSWV and tomato chlorotic spot virus infected tomato fruits. It further showed that TSWV resistance breaking in YNAU335 and disease development in fruit may not be related to tomato chlorotic spot virus, but related to TSWV.

Disease synergism in pepper (*Capsicum annuum*) plants harboring the *Tsw* resistance gene was found after simultaneous inoculation of resistance-breaking (RB) and non-resistance-breaking (NRB) isolates [40]. Co-infection of sweet orange with severe (CTV-B6) and mild (CTV-B2) strains of the *citrus tristeza* virus was found to be overwhelmingly dominated by the severe strain, which shows that CTV-B2 does not provide a useful level of cross-protection to citrus against CTV-B6 [41]. In this study, the NP and MP of TSWV NRB and RB isolates were cloned from tomato fruits. Our results showed that the severity of the disease in tomato fruits was much greater than that in previous studies in terms of the degree of disease in a single fruit and the number of diseased fruits. This suggested that large-scale disease in tomato fruit was related to NRB and RB isolates infecting the fruit.

Morphological characteristics observation and small RNA sequencing showed that the amount of other viruses in the fruit of YNAU335 was significantly lower than that of TSWV. Combined with the typical symptoms of TSWV on the surface of the fruit, we deduced that the susceptibility of the fruit was mainly due to TSWV infection. We compared the mutations found in the NP and MP of TSWV. A total of 37 positive clones from the MP and 36 from the NP were selected for sequencing and compared with the amino acid sequences of the MPs (GenBank MK887284) and NPs (GenBank MK628737, MK628738, and MK628739) from resistance-breaking isolates that were found in previous studies. This result revealed rich genetic diversity in the MP and, in particular, the NP of the tomato fruit. Based on a combination of their frequencies, the amino acid mutations, especially that at F118 in the MP, were suggested to play a major role in YNAU335 resistance-breaking by TSWV. A previous study showed that the mutation at F118 from C to Y and mutation T120 to N were related to *Sw-5* resistance-breaking [29]. This result further suggests that AA118 in MP shows rich polymorphism, and mutations at this site can change the infection characteristics of TSWV. One quality gene *SlCHS3* was successfully mapped in YNAU335, which conferred immunity to TSWV [30]. However, the mechanism between the mutation F at AA118 of the MP and the breaking of the *SlCHS3′*s resistance needs to be further verified.

## 5. Conclusions

The immune characteristic of tomato inbred line YNAU335 to TSWV was broken, and the disease symptoms only occurred on fruit. In addition to other plant viruses, TSWV accounted for the highest proportion of infected fruits. Mutation F at AA118 of the MP, developing only in the infected fruit, was likely to be the key to breaking the resistance to TSWV.

## Figures and Tables

**Table 1:** Differences among the amino acid sequences of the movement protein in the TSWV-positive clones. The new mutant bases are colored red. MK883723 and MK883724 are derived from non-resistance-breaking isolates of TSWV. MK887284 is derived from resistance-breaking isolates of TSWV.

Positive Clones	Amino Acid Position										
	42	53	65	86	115	118	124	157	175	201	274
MK883723	E	S	S	R	I	C	R	M	I	V	E
MK883724, MP-2, MP-4, MP-5, MP-9, MP-12, MP-16, MP-20, MP-22, MP-24, MP-25, MP-31, MP-32, MP-33, MP-34, MP-35, MP-36										M	
MK887284										M	K
MP-1		**P**								M	
MP-3, MP-6, MP-7, MP-10, MP-13, MP-18, MP-19, MP-21, MP-23, MP-28, MP-29, MP-30, MP-37						**F**				M	
MP-8					**T**					M	
MP-11				**G**						M	
MP-14	**G**									M	
MP-15						**F**		**T**		M	
MP-17		**P**				**F**			**V**	M	

**Table 2:** Differences among the amino acid sequences of the nucleocapsid protein of the TSWV positive clones. The new mutant bases are colored red. MK628735 and MK628736 are derived from non-resistance-breaking isolates of TSWV. MK628737, MK628738 and MK628739 are derived from resistance-breaking isolates of TSWV.

Positive Clones	Amino Acid Position														
	10	18	35	36	39	100	117	135	137	167	176	210	213	248	255
MK628735, NP-1, NP-4, NP-6, NP-10, NP-13, NP-15, NP-20, NP-21, NP-23, NP-24, NP-26, NP-28, NP-34, NP-35, NP-36	N	G	K	T	L	I	I	D	A	A	D	K	K	V	T
MK628736, NP-8, NP-9, NP-11, NP-14, NP-16, NP-25, NP-29, NP-30, NP-31, NP-32															A
MK628737					R										
MK628738				I											
MK628739		V													A
NP-2												**E**			
NP-3				**A**				**N**							A
NP-5														**I**	
NP-7						**F**									
NP-12									**T**						A
NP-17											**G**				
NP-18								**N**					**E**		A
NP-19										**T**					
NP-22			**E**												
NP-27							**V**								
NP-33	**T**														A

## Data Availability

Not applicable.

## Supplementary Materials

The following supporting information can be downloaded at: https://www.mdpi.com/article/10.3390/genes14091788/s1, Table S1: Primers used for RT-PCR; Table S2: The virus species in fruits of YNAU335 planted in 2014 to 2017. The yellow shading shows plant viruses; Table S3: The virus species in leaves of YNAU335 planted in 2014 to 2017. The yellow shading shows plant viruses; Table S4: The virus species in fruits of YNAU335 planted in 2018 to 2020. The yellow shading shows plant viruses; Table S5: The virus species in leaves of YNAU335 planted in 2018 to 2020. The yellow shading shows plant viruses; Table S6: The virus species in fruits of ynau335 planted in 2021 to 2022. The yellow shading shows plant viruses; Table S7: The virus species in leaves of ynau335 planted in 2021 to 2022. The yellow shading shows plant viruses.
