# Supplementary material for: Pathological Features and Genetic Polymorphism Analysis of Tomato Spotted Wilt Virus in Infected Tomato Fruit
Source: Genes (Basel). 2023 Sep 12;14(9):1788. doi: 10.3390/genes14091788 (PMC10531454; doi:10.3390/genes14091788)
Supplement: Supplementary file 1 [file genes-14-01788-s001.zip › genes-2596143-supplementary/Supplementary File/Table S1.pdf]

Table S1 Primers used for RT-PCR

Primer names	Primer sequences (5'→3')	
pMP	Forward	ATGTTGACTTTTTTTGGTAATAAGG
	Reverse	CTATATTTCATCAAAAGATAACTGAGC
pNP	Forward	TGTCTA AGGTT AAGCTCACTAAG
	Reverse	TYAAGCAAGTTCTGYGAGTTTTGCC
pTZSV	Forward	ATGAGGAGAACAAGGCTAA
	Reverse	GAATGTCAGTCTGTAGCA
pToBRFV	Forward	GAAGTCCCGATGTCTGTAAGG
	Reverse	GTGCCTACGGATGTGTATGA
