# Supplementary material for: Pathological Features and Genetic Polymorphism Analysis of Tomato Spotted Wilt Virus in Infected Tomato Fruit
Source: Genes (Basel). 2023 Sep 12;14(9):1788. doi: 10.3390/genes14091788 (PMC10531454; doi:10.3390/genes14091788)
Supplement: Supplementary file 1 [file genes-14-01788-s001.zip › genes-2596143-supplementary/Supplementary File/Table S2.pdf]

Table S2 The virus species in fruits of YNAU335 planted in 2014 to 2017 using small RNA sequencing. The yellow shading shows plant viruses.

NO.	Virus species annotated to the virus database	The number of sequences aligned to the virus	The rate of sequences aligned to the virus in all the sequences aligned to the virus database
1	Pepper chlorotic spot virus	1310	23.68%
2	Oxbow virus	1310	23.68%
3	Shamonda orthobunyavirus	890	16.09%
4	Choristoneura occidentalis granulovirus	636	11.50%
5	Enterobacteria phage DE3	626	11.31%
6	Enterobacteria phage 13a	90	1.63%
7	Escherichia phage 64795_ec1	90	1.63%
8	Escherichia phage CICC 80001	90	1.63%
9	Yersinia pestis phage phiA1122	90	1.63%
10	Stenotrophomonas phage IME15	90	1.63%
11	Enterobacteria phage T7	90	1.63%
12	Tadarida brasiliensis circovirus 1	83	1.50%
13	Bat associated circovirus 1	83	1.50%
14	Tobacco vein clearing virus	43	0.78%
15	Southern tomato virus	10	0.18%
