# Supplementary material for: Pathological Features and Genetic Polymorphism Analysis of Tomato Spotted Wilt Virus in Infected Tomato Fruit
Source: Genes (Basel). 2023 Sep 12;14(9):1788. doi: 10.3390/genes14091788 (PMC10531454; doi:10.3390/genes14091788)
Supplement: Supplementary file 1 [file genes-14-01788-s001.zip › genes-2596143-supplementary/Supplementary File/Table S3.pdf]

Table S3 The virus species in leaves of YNAU335 planted in 2014 to 2017 using small RNA sequencing . The yellow shading shows plant viruses.

NO.	Virus species annotated to the virus database	The number of sequences aligned to the virus	The rate of sequences aligned to the virus in all the sequences aligned to the virus database
1	Pepper chlorotic spot virus	1509	33.34%
2	Oxbow virus	1509	33.34%
3	Choristoneura occidentalis granulovirus	452	9.99%
4	Shamonda orthobunyavirus	369	8.15%
5	Enterobacteria phage DE3	169	3.73%
6	Tobacco vein clearing virus	82	1.81%
7	Bat associated circovirus 1	62	1.37%
8	Tadarida brasiliensis circovirus 1	62	1.37%
9	Yersinia pestis phage phiA1122	52	1.15%
10	Enterobacteria phage T7	52	1.15%
11	Escherichia phage CICC 80001	52	1.15%
12	Stenotrophomonas phage IME15	52	1.15%
13	Escherichia phage 64795_ec1	52	1.15%
14	Enterobacteria phage 13a	52	1.15%
