# Supplementary material for: Pathological Features and Genetic Polymorphism Analysis of Tomato Spotted Wilt Virus in Infected Tomato Fruit
Source: Genes (Basel). 2023 Sep 12;14(9):1788. doi: 10.3390/genes14091788 (PMC10531454; doi:10.3390/genes14091788)
Supplement: Supplementary file 1 [file genes-14-01788-s001.zip › genes-2596143-supplementary/Supplementary File/Table S4.pdf]

Table S4 The virus species in fruits of YNAU335 planted in 2018 to 2020 using small RNA sequencing. The yellow shading shows plant viruses.

NO.	Virus species annotated to the virus database	The number of sequences aligned to the virus	The rate of sequences aligned to the virus in all the sequences aligned to the virus database
1	Tomato spotted wilt virus	81908	74.53%
2	Tomato chlorotic spot virus	15627	14.22%
3	Zucchini lethal chlorosis virus	4356	3.96%
4	Chrysanthemum stem necrosis virus	2740	2.49%
5	Groundnut ringspot and Tomato chlorotic spot virus reassortant	2621	2.38%
6	Groundnut ringspot virus	687	0.63%
7	Melon severe mosaic tospovirus	620	0.56%
8	Choristoneura occidentalis granulovirus	367	0.33%
9	Pepper chlorotic spot virus	296	0.27%
10	Oxbow virus	296	0.27%
11	Southern tomato virus	206	0.19%
12	Tobacco vein clearing virus	76	0.07%
13	Escherichia phage CICC 80001	24	0.02%
14	Enterobacteria phage T7	24	0.02%
15	Yersinia pestis phage phiA1122	24	0.02%
16	Enterobacteria phage 13a	24	0.02%
