# Supplementary material for: Pathological Features and Genetic Polymorphism Analysis of Tomato Spotted Wilt Virus in Infected Tomato Fruit
Source: Genes (Basel). 2023 Sep 12;14(9):1788. doi: 10.3390/genes14091788 (PMC10531454; doi:10.3390/genes14091788)
Supplement: Supplementary file 1 [file genes-14-01788-s001.zip › genes-2596143-supplementary/Supplementary File/Table S5.pdf]

Table S5 The virus species in leaves of YNAU335 planted in 2018 to 2020 usingsmall RNA sequencing. The yellow shading shows plant viruses.

NO.	Virus species annotated to the virus database	The number of sequences aligned to the virus	The rate of sequences aligned to the virus in all the sequences aligned to the virus database
1	Tomato spotted wilt virus	19316	49.69%
2	Tomato chlorotic spot virus	7434	19.12%
3	Groundnut ringspot and Tomato chlorotic spot virus reassortant	2283	5.87%
4	Zucchini lethal chlorosis virus	1960	5.04%
5	Chrysanthemum stem necrosis virus	1251	3.22%
6	Southern tomato virus	1193	3.07%
7	Oxbow virus	1147	2.95%
8	Pepper chlorotic spot virus	1131	2.91%
9	Choristoneura occidentalis granulovirus	874	2.25%
10	Groundnut ringspot virus	409	1.05%
11	Melon severe mosaic tospovirus	358	0.92%
12	Bat associated circovirus 1	351	0.90%
13	Tadarida brasiliensis circovirus 1	351	0.90%
14	Enterobacteria phage DE3	272	0.70%
15	Escherichia phage CICC 80001	91	0.23%
16	Escherichia phage 64795_ec1	91	0.23%
17	Yersinia pestis phage phiA1122	91	0.23%
18	Stenotrophomonas phage IME15	91	0.23%
19	Enterobacteria phage 13a	91	0.23%
20	Enterobacteria phage T7	91	0.23%
