# Supplementary material for: Pathological Features and Genetic Polymorphism Analysis of Tomato Spotted Wilt Virus in Infected Tomato Fruit
Source: Genes (Basel). 2023 Sep 12;14(9):1788. doi: 10.3390/genes14091788 (PMC10531454; doi:10.3390/genes14091788)
Supplement: Supplementary file 1 [file genes-14-01788-s001.zip › genes-2596143-supplementary/Supplementary File/Table S6.pdf]

Table S6 The virus species in fruits of YNAU335 planted in 2021 to 2022 using small RNA sequencing. The yellow shading shows plant viruses.

NO.	Virus species annotated to the virus database	The number of sequences aligned to the virus	The rate of sequences aligned to the virus in all the sequences aligned to the virus database
1	Tomato spotted wilt virus	62223	68.44%
2	Shamonda orthobunyavirus	5731	6.30%
3	Tomato chlorotic spot virus	5226	5.75%
4	Zucchini lethal chlorosis virus	5048	5.55%
5	Chrysanthemum stem necrosis virus	2584	2.84%
6	Groundnut ringspot and Tomato chlorotic spot virus reassortant	2295	2.52%
7	Choristoneura occidentalis granulovirus	1921	2.11%
8	Groundnut ringspot virus	914	1.01%
9	Oxbow virus	855	0.94%
10	Pepper chlorotic spot virus	679	0.75%
11	Enterobacteria phage DE3	532	0.59%
12	Diolcogaster facetosa bracovirus	471	0.52%
13	Tadarida brasiliensis circovirus 1	426	0.47%
14	Bat associated circovirus 1	426	0.47%
15	Alstroemeria yellow spot virus	375	0.41%
16	Melon severe mosaic tospovirus	375	0.41%
17	Southern tomato virus	255	0.28%
18	Enterobacteria phage T7	131	0.14%
19	Escherichia phage CICC 80001	74	0.08%
20	Yersinia pestis phage phiA1122	74	0.08%
21	Escherichia phage 64795_ec1	74	0.08%
22	Stenotrophomonas phage IME15	74	0.08%
23	Enterobacteria phage 13a	74	0.08%
24	Tobacco vein clearing virus	59	0.06%
25	Trichoplusia ni ascovirus 2c	20	0.02%
