# Supplementary material for: Pathological Features and Genetic Polymorphism Analysis of Tomato Spotted Wilt Virus in Infected Tomato Fruit
Source: Genes (Basel). 2023 Sep 12;14(9):1788. doi: 10.3390/genes14091788 (PMC10531454; doi:10.3390/genes14091788)
Supplement: Supplementary file 1 [file genes-14-01788-s001.zip › genes-2596143-supplementary/Supplementary File/Table S7.pdf]

Table S7 The virus species in leaves of YNAU335 planted in 2021 to 2022 using small RNA sequencing. The yellow shading shows plant viruses.

NO.	Virus species annotated to the virus database	The number of sequences aligned to the virus	The rate of sequences aligned to the virus in all the sequences aligned to the virus database
1	Pepper chlorotic spot virus	2942	26.84%
2	Southern tomato virus	2172	19.82%
3	Oxbow virus	1515	13.82%
4	Choristoneura occidentalis granulovirus	955	8.71%
5	Enterobacteria phage DE3	840	7.66%
6	Vibrio phage Thalassa	466	4.25%
7	Vibrio phage Ceto	466	4.25%
8	Vibrio phage pVp-1	466	4.25%
9	Tobacco vein clearing virus	239	2.18%
10	Enterobacteria phage T7	170	1.55%
11	Escherichia phage CICC 80001	141	1.29%
12	Yersinia pestis phage phiA1122	141	1.29%
13	Enterobacteria phage 13a	141	1.29%
14	Escherichia phage 64795_ec1	141	1.29%
15	Stenotrophomonas phage IME15	141	1.29%
16	Tokyovirus A1	25	0.23%
